# Association between interleukin-6 gene polymorphisms and febrile seizure risk: A meta-analysis

**DOI:** 10.1097/MD.0000000000017167

**Published:** 2019-09-27

**Authors:** Qingling Chen, Mengmeng Li, Xin Zhang, Xinyue Zhang, Rui Zhong, Weihong Lin

**Affiliations:** a Department of Neurology; b Department of Hepatology, The First Hospital of Jilin University, Chang Chun, Ji Lin Province, China.

**Keywords:** febrile seizure risk, interleukin-6 gene, meta-analysis

## Abstract

**Background:**

The association between plasma interleukin-6 (IL-6) levels and the development of febrile seizures (FS) has been reported in multiple previous studies, which showed significantly higher serum IL-6 levels in FS patients than in control patients. However, the mechanism underlying this association remains unclear. One previous study indicated an increased frequency of the −174 GG and −597 GG genotypes in FS patients. Although IL-6 gene polymorphisms may be associated with FS risk, this association remains a matter of debate.

**Objective:**

Considering the lack of meta-analyses addressing the possible association between IL-6 gene polymorphisms and the risk of FS, we aimed to perform a meta-analysis to determine the association of IL-6 gene polymorphisms (−572, −174, −597) with the risk of FS.

**Methods:**

We conducted a systematic literature search in the PubMed, EMBASE, and WANFANG databases to collect eligible articles. The associations of IL-6 gene polymorphisms with FS risk were evaluated by calculating the pooled odds ratios and 95% confidence intervals. The dominant, recessive, heterozygous, homozygous, and allele genetic models were used to calculate the combined ORs.

**Results:**

Our meta-analysis showed that IL-6 (−572, −174, −597) polymorphisms were significantly associated with susceptibility to FS.

**Conclusion:**

This study provided knowledge regarding the association of IL-6 (572, 174, 597) polymorphisms with susceptibility to FS. The T allele and TT genotype may be associated with an increased risk for FS.

## 1 Introduction

Febrile seizures (FS), the most common seizures suffered by children in early childhood, occur in 2% to 5% of infants and children aged 6 months to 5 years. FS are defined as seizures accompanied by fever without intracranial infection, hypoglycemia, or acute electrolyte imbalance; they can be classified as simple FS (which have a duration of less than 15 minutes) and complex febrile FS (which last for more than 15 minutes).^[1,2]^

One previous investigation showed that neuronal excitability can be enhanced by certain cytokines, which may play an important role in FS.^[3]^ Interleukin-6 (IL-6) is one such proinflammatory cytokine that shows biological activity in inflammation and is associated with fever in acute-stage FS. The association of IL-6 level in the plasma and cerebrospinal fluid with the development of FS has been reported in previous studies for years. Activation and release of IL-6 are believed to cause fever in FS patients. Azab et al reported significantly higher serum IL-6 levels in FS patients in comparison with those in control patients in their study, which was partly supported by the results of other studies.^[4–6]^ Kalueff et al^[7]^ indicated that exogenously administered IL-6 has strong proconvulsive effects in a seizure model in rats.

The mechanism underlying the presence of higher serum IL-6 levels in FS remains unclear. However, considering the importance of IL-6, previous studies have evaluated the role of IL-6 single-nucleotide polymorphisms (SNPs) in groups of FS patients. Although IL-6 gene polymorphisms may be associated with FS risk, the presence of such an association remains a matter of debate. Previous studies exploring the associations of FS with IL-6 gene polymorphisms showed an increased frequency of the −174 GG and −597 GG genotypes in FS patients, indicating that the −174 and −597 polymorphisms in the IL-6 gene were significantly associated with FS, whereas the −572 polymorphism was not significantly associated with the risk of FS.^[8]^ However, Nur et al^[9]^ reported significant associations between FS risk and IL-6 −174 GG and −572 GG genotypes. Thus, the results in different studies have been debatable, and even conflicting. In addition, the associations of IL-6 gene polymorphisms may differ across patients of different ethnicities. Nevertheless, no previous meta-analysis has assessed these aspects. Therefore, we performed a meta-analysis to determine the association of IL-6 gene polymorphisms with the risk of FS.

## 2 Materials and methods

Ethical approval was not necessary, and all analyses in the present study were based on previous published studies, thus no ethical approval and patient consent are required.

### 2.1 Literature search

This meta-analysis was performed according to the PRISMA guideline. We conducted a systematic literature search in the PubMed, EMBASE, and WANFANG database to collect eligible articles using the following combinations of PubMed keywords from inception to November 1, 2018: (“febrile seizure” OR “seizure, fever” OR “seizure, febrile”) AND (“interleukin-6” OR “interleukin 6” OR “IL-6” OR “IL 6”) AND (“polymorphism” OR “genetic polymorphism” OR “SNP” OR “variant”). The reference lists of the included studies were also screened to identify additional articles. We restricted our search to case-control trials for meta-analysis. Only English and Chinese articles were included.

### 2.2 Study selection

The studies included in the meta-analysis fulfilled the following inclusion criteria: case-control design; studies evaluating the associations of IL-6 gene polymorphisms with FS risk; and FS diagnosed on the basis of standard criteria; and studies reporting genotype or allele frequencies in both cases and controls. Accordingly, the exclusion criteria were as follows: duplicates; case reports, reviews, letters, or meta-analysis; studies with inadequate information; and studies with overlapping data. We first screened the titles and abstracts to exclude studies that obviously did not fulfil the inclusion criteria. The remaining studies were assessed for eligibility by examining the full text. Two reviewers (Zhong and Chen) independently checked the articles and resolved disagreements by discussion. Only the most relevant articles were included in the final analysis.

### 2.3 Quality assessment

Two reviewers independently assessed the quality of each included study on the basis of the Newcastle–Ottawa scale. Trials with a score of at least 6 stars were considered to be of good quality, and those rated below 6 stars were classified as poor-quality trials. Disagreements were resolved by discussion or by the third reviewer to ensure a consistent outcome.

### 2.4 Date abstraction

Two reviewers independently extracted primary data from the selected studies. The following information was extracted from each study: first author, year of publication, country, ethnicity, study design, number of cases and controls, gene polymorphism data, allele frequencies and genotype distributions of cases and controls, and Hardy–Weinberg equilibrium (HWE) among controls. When this information was incomplete, we checked supplementary data and contacted the corresponding authors for the required data.

### 2.5 Statistical analysis

The associations of IL-6 gene polymorphisms with FS risk were evaluated by calculating the pooled odds ratios (ORs) and 95% confidence intervals (CIs). The dominant, recessive, heterozygous, homozygous, and allele genetic models were used to calculate the combined ORs. Heterogeneity was assessed on the basis of the *P* values of the chi-square and *I*^2^ statistics, and heterogeneity was significant if the *I*^2^ statistic was greater than 50% or the *P* value was less than .1. We used a randomized-effects model to pool the results with significant heterogeneity in our meta-analysis; otherwise, a fixed-effects model was applied. To explore the potential source of heterogeneity, subgroup analysis was carried out on the basis of ethnicity (Caucasians vs Asians). In addition, sensitivity analysis was conducted by removing 1 study at a time to evaluate the stability of the results. We performed Begg test and Egger test to assess publication bias (*P* < .05 was considered statistically significant). All statistical analyses were conducted with the software STATA 12.0. *P* values <.05 indicated statistical significance.

## 3 Results

### 3.1 Study selection

**Figure 1:**
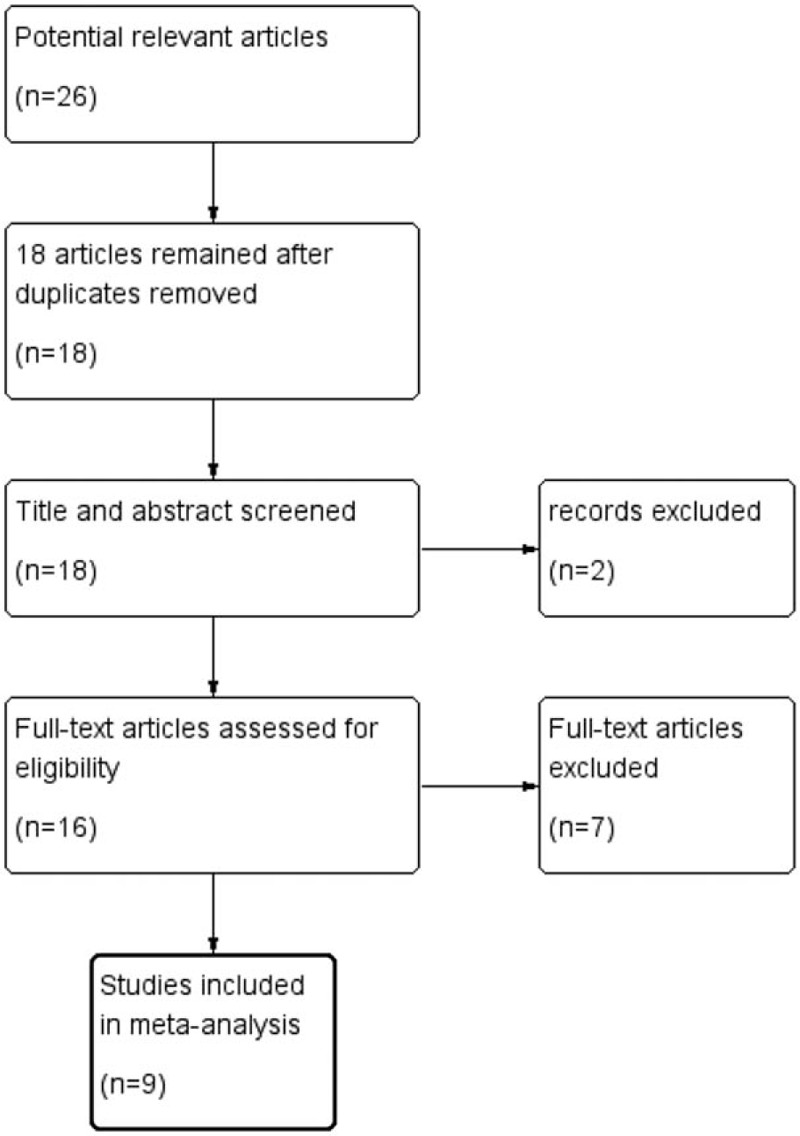
The process of identification of studies.

The search strategy yielded 26 references, of 18 articles remained after removing 8 duplicates. We excluded 2 articles that were obviously irrelevant, and the full text of the remaining 16 articles was assessed in detail. After full-text assessment, we eventually identified 9 studies^[8–16]^ that could be used for the meta-analysis. A flowchart of the process used for identification of studies is presented in Fig. 1.

We eventually included 9 case-control trials with 2091 individuals (1017 FS cases and 1074 controls) in the meta-analysis, and the median number of patients per study was 232 (range 40–237). The 9 studies were performed in 5 countries (number of studies): China (4, including 1 study in Taiwan); Japan (2); Iran (1); Italy (1); and Turkey (1). The ethnicities of the participants in these studies were varied, including Asian (7) and Caucasian (2). Eight studies reported the genotype distributions of the IL-6 (−572) polymorphism in FS cases and controls. Six of these studies were performed in Asian participants and 2 were performed in Caucasians. Five studies investigated the genotype distributions of the IL-6 (−174) polymorphism and 1 study was excluded due to insufficient data. Three studies reported the genotype distributions of the IL-6 (−592) polymorphism. The characteristics of the included studies are listed in Table 1. The IL-6 (−572, −174, −592) genotypes and allele distribution among FS cases and controls are summarized in Table 2. Genotype values in the control group were in agreement with the HWE in 6 studies. The 9 trials were evaluated using the Newcastle–Ottawa scale, and the results are shown in Table 1. Three studies scored 8, 2 scored 7, and 4 scored 6, indicating good quality.

**Table 1:**
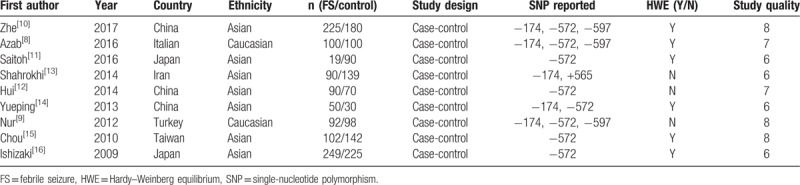
The characteristics of included studies.

**Table 2:**
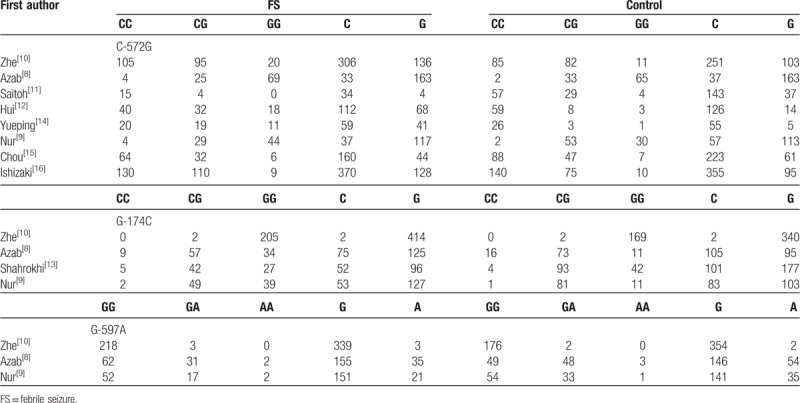
Interleukin-6 (IL-6) (−572, −174, −592) genotype and allele distribution among FS cases and controls.

### 3.2 Characteristics of the studies and quality assessment

#### 3.2.1 Meta-analysis

##### 3.2.1.1 IL-6 (−572) polymorphism and FS risk

**Figure 2:**
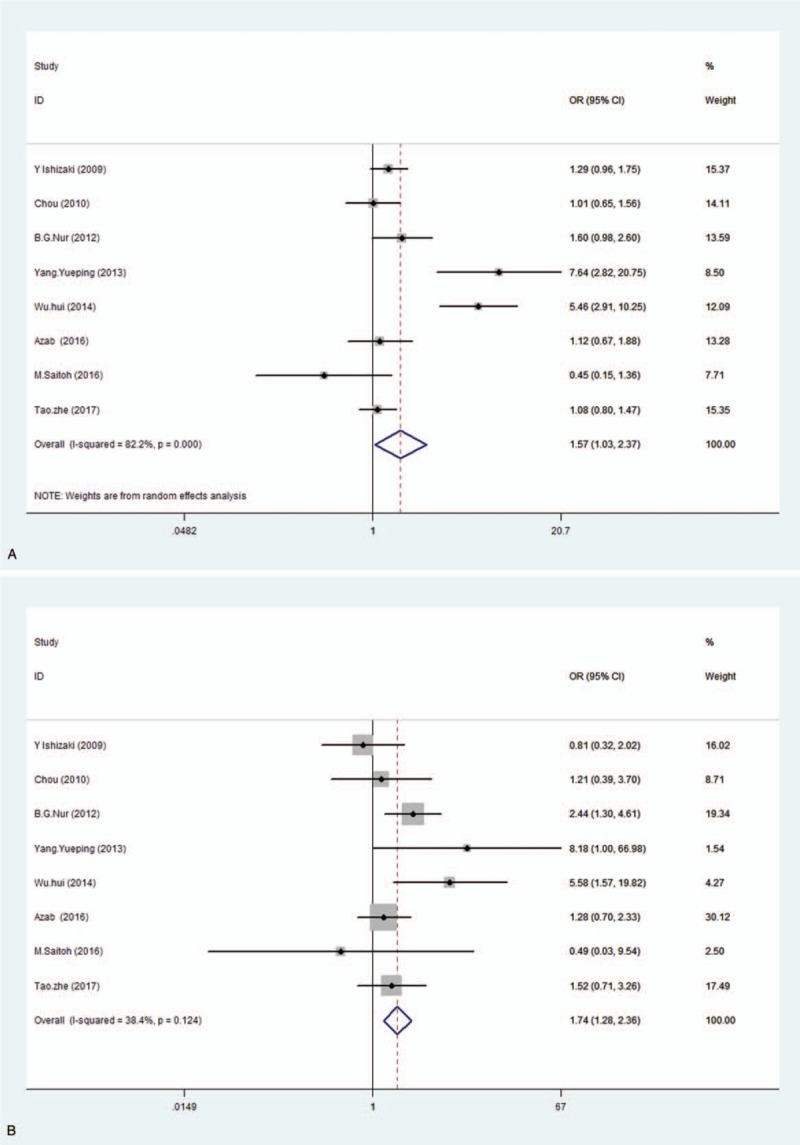
Forest plots of odds ratios (ORs) and 95% confidence intervals (CIs) for the association between the IL-6 (−572) polymorphism and FS risk in the allele model (A) and the recessive model (B).

The association between the IL-6 (−572) polymorphism and FS risk was assessed in 8 studies. The random-effect model was used due to the significant heterogeneity. The pooled results indicated that the frequency of G allele was significantly higher in FS group than controls in the allele model. In addition, individuals carrying GG genotype had an increased risk for the development of FS when compared with (CG + CC) genotype in recessive models (G vs C: OR 1.57, 95% CI 1.03–2.37; GG vs [CG + CC]: OR 1.735, 95% CI 1.277–2.357), but we did not observe a positive association in the other genetic models. We conducted subgroup analyses for ethnicity in all genetic models to explore the potential sources of heterogeneity. The heterogeneity still persisted in the Asian group, but it significantly decreased in the Caucasian group after the analysis. The recessive model showed a significant association of the IL-6 (−572) polymorphism with FS in Asians (GG vs [CG + CC]: OR 1.735, 95% CI 1.124–2.678). However, this association was not present in Caucasians. The results are presented in Table 3 and depicted using forest plots in Fig. 2. The sensitivity analysis yielded statistically robust results. The Begg test and Egger test did not show a significant publication bias (*P* > .05).

**Table 3:**
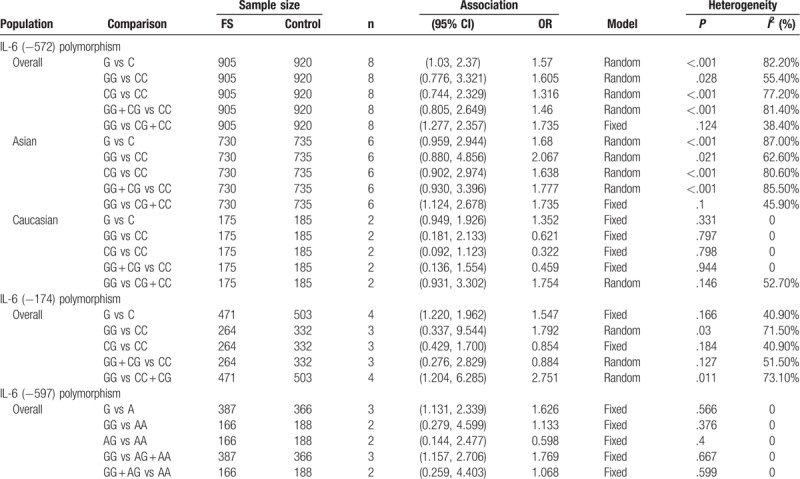
Summary of pooled odds ratios (ORs) with 95% confidence interval (95% CIs) in the meta-analysis.

##### 3.2.1.2 IL-6 (−174, −597) polymorphisms and FS risk

**Figure 3:**
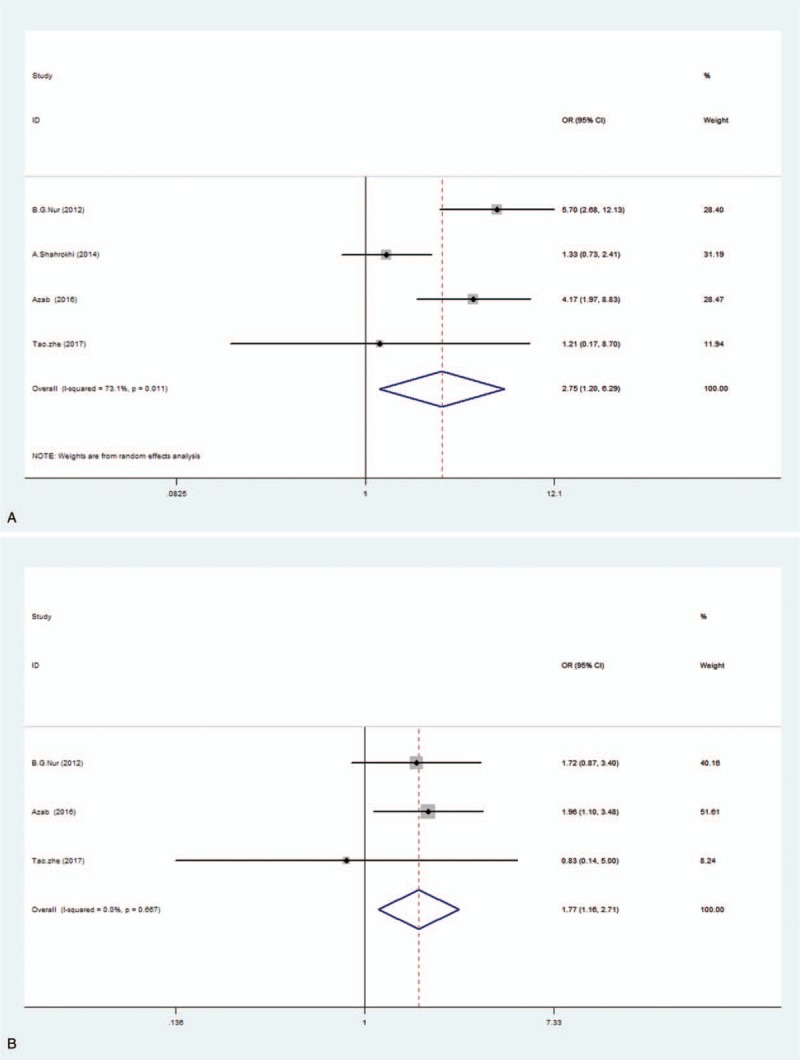
Forest plots of odds ratios (ORs) and 95% confidence intervals (CIs) for the association between IL-6 (−174, −597) polymorphisms and FS risk in the recessive model (A, B).

We identified 4 studies assessing the association between the IL-6 (−174) polymorphism and FS susceptibility, and a total of 471 FS cases and 503 controls were included. Our meta-analysis by polling the results showed a significant association of the IL-6 (−174) polymorphism with FS risk in the allele and recessive models (G vs C: OR 1.547, 95% CI 1.220–1.962; GG vs [CG + CC]: OR 2.751, 95% CI 1.204–6.285) (Table 3), which were shown as forest plots (Fig. 3). In other words, we found individuals with GG genotype have significantly more susceptibility to FS than those with CG or CC, and individuals with G allele have significantly more susceptibility to FS than those with C allele. Similarly, our meta-analysis yielded evidence for a significant association between the IL-6 (−597) polymorphism and susceptibility to FS in the allele and recessive models (G vs A: OR 1.626, 95% CI 1.131–2.339; GG vs [AG + AA]: OR 1.769, 95% CI 1.157–2.706) (Table 3). The results are presented in Table 3 and depicted as forest plots in Fig. 3.

## 4 Discussion

The relationship between high plasma IL-6 levels and FS has been well-established.^[5]^ The activation and release of IL-6 can cause fever in FS patients, and increased fever is a significant independent risk factor for patients with a history of FS.^[17]^ IL-6 can regulate the central thermoregulatory mechanisms in the preoptic area of the hypothalamus and lead to fever.^[18]^ The IL-6 gene is located on chromosomes 7p21, and IL-6 gene polymorphism has been found to be associated with the high plasma IL-6 levels, which can cause many diseases. Ferrari et al^[19]^ found that the IL-6 (−572) polymorphism can influence the gene expression level. In another study, the presence of the IL-6 (−174) G allele was associated with higher IL-6 protein levels in comparison with those obtained with the C allele.^[20]^ The IL-6 (−572) SNP was proven to be associated with several diseases by significantly increasing IL-6 expression, such as chronic kidney disease,^[21]^ acute coronary syndrome,^[22]^ and cerebral infarction.^[23]^

The role of specific IL alleles associated with FS has been extensively studied. The findings of a recent meta-analysis showed a possible association of the IL-1 rs16944 polymorphism with the risk of FS.^[24]^ Our study aimed to provide more evidence for the associations between specific IL alleles and the risk of FS. Our meta-analysis found that the IL-6 (−572, −174, −597) polymorphisms may be significantly associated with susceptibility to FS. We observed a significant association between FS risk and the G allele and the recessive homozygote GG in the −572 polymorphism, suggesting that this gene polymorphism may contribute to FS development. The frequency of G allele was significantly higher in FS group than controls in the allele model. In addition, individuals carrying GG genotype had an increased risk for the development of FS when compared with (CG + CC) genotype in recessive models. In subgroup analysis, to address the differences in allele frequency attributable to ethnicity, a significant association was observed only in Asians, but not in Caucasian patients. The findings of our meta-analysis revealed a significant relationship between the −572 polymorphism and FS susceptibility in the allele and recessive models. However, we observed significant heterogeneity among studies, and after subgroup analyses, the heterogeneity persisted among patients. For the IL-6 (−174) polymorphism, individuals carrying the G allele showed a greater risk for FS than those carrying the C allele, and individuals with the GG genotype were more susceptible to FS than those with the CC + CG genotype. Similarly, for the IL-6 (−597) polymorphism, we observed a positive association between the G allele and FS susceptibility, and the GG genotype was associated with a high risk of FS.

The association of the IL-6 polymorphisms with susceptibility to FS has been studied extensively, especially with respect to the IL-6 (−572) polymorphism. Previous studies have tried to clarify the role of this polymorphism in the development of FS. Ishizaki et al first reported the IL-6 (−572) polymorphism, but they did not find any association between this polymorphism and FS risk. Several subsequent studies obtained similar findings.^[8,15]^ However, the results obtained by Nur et al contradicted those obtained previously, and their study revealed that the frequency of the GG genotype was positively associated with FS risk, which was supported by the results of studies from China.^[12,14]^ These inconsistencies in the findings of previous studies may be attributable to several factors, including inadequate sample sizes and differences in allele frequencies across different ethnic backgrounds. Our comprehensive meta-analysis with different genetic models aimed to better testify the association between IL-6 polymorphisms and FS susceptibility. Our meta-analysis included 9 studies and may have more adequate statistical power to yield a precise conclusion. Furthermore, we also conducted subgroup analyses based on ethnicity to address the differences in allele frequency attributable to ethnicity. The findings of our meta-analysis revealed a significant relationship between the −572 polymorphism and FS susceptibility in the allele and recessive models. However, we observed significant heterogeneity among studies, and after subgroup analyses, the heterogeneity persisted in the Asian group, but significantly decreased in the Caucasian group. This heterogeneity may have led to the different allele and genotype frequencies in different studies. We performed sensitivity analysis to evaluate the influence of each study on the overall OR, indicating the stability of overall ORs.

To the best of our knowledge, our meta-analysis is the first study to pool the results of case-control trials to assess the association of IL-6 (−572, −174, −597) polymorphisms with susceptibility to FS. Our work further certified the association between IL-6 (−572, −174, −597) polymorphisms and FS risk. However, the results should be interpreted with caution, because they were not sufficient to draw a conclusion. Additional molecular studies are required to confirm our findings. In addition, several limitations in the meta-analysis should be acknowledged. First, a meta-analysis may be biased when the literature search fails to identify all relevant studies. However, access to unpublished articles remains difficult, which might be a potential limitation of our study. Nevertheless, assessment of the publication bias did not yield any significant publication bias in our study. Second, the number of included studies was limited, and the sample size was small. Thus, there is a need to conduct more trials to confirm the findings of this meta-analysis. Third, we did not evaluate gene–gene and gene–environment interactions. Fourth, subgroup analysis based on the age, sex, or subtype of FS was not performed. Thus, future studies with large sample sizes and detailed characteristics are required. In addition, we did not branch out other state analysis methods such as deep learning-based studies in our meta-analysis.

## 5 Conclusions

In conclusion, this study provided knowledge regarding the association of IL-6 (−572, −174, −597) polymorphisms with susceptibility to FS. The T allele and TT genotype may be associated with an increased risk for FS. Further large-scale studies with consideration of gene–gene and gene–environment interactions are required to evaluate the causal mechanism underlying this association.

## Author contributions

**Conceptualization:** Rui Zhong.

**Data curation:** Rui Zhong.

**Formal analysis:** Xin Zhang.

**Investigation:** Rui Zhong, Qingling Chen, Weihong Lin.

**Methodology:** Rui Zhong, Qingling Chen, Mengmeng Li.

**Resources:** Mengmeng Li, Xin Zhang.

**Software:** Rui Zhong, Qingling Chen, Mengmeng Li, Xinyue Zhang.

**Supervision:** Rui Zhong, Qingling Chen, Xinyue Zhang, Weihong Lin.

**Validation:** Rui Zhong, Qingling Chen, Mengmeng Li, Weihong Lin.

**Visualization:** Rui Zhong, Xin Zhang, Xinyue Zhang.

**Writing – original draft:** Rui Zhong, Qingling Chen, Xin Zhang, Xinyue Zhang.

**Writing – review & editing:** Rui Zhong, Qingling Chen, Weihong Lin.
